# Analysis of related factors for pathological upgrading of cervical biopsy from CIN3 to cancer after conical resection

**DOI:** 10.1186/s12885-024-12186-w

**Published:** 2024-04-01

**Authors:** Zhifang Li, Guiju Zhou, Longfan Jiang, Mengjie Wang

**Affiliations:** 1 Department of Gynaecology, Anqing Municipal Hospital, Anhui Medical University, Anqing, Anhui Province P.R. China; 2 The Second Affiliated Hospital of Anhui Medical University, Anqing, Anhui Province P.R. China

**Keywords:** Cervical intraepithelial neoplasia, Biopsy, Uterine cervical neoplasms, Pathological upgrading, Risk factors

## Abstract

**Background:**

To investigate related factors for postoperative pathological upgrading of cervical biopsy to cervical cancer (CC) in patients with cervical intraepithelial neoplasia (CIN)3 after conical resection.

**Methods:**

This retrospective study collected data from patients diagnosed with CIN3 by cervical biopsies at the author’s Hospital between January 2012 and December 2022. The primary outcome was the pathological results of patients after conical resection. The pathological findings were categorized into the pathological upgrading group if postoperative pathology indicated CC, while those with normal, inflammatory, or cervical precancerous lesions were classified into the pathological non-upgrading group. The factors associated with upgrading were identified using multivariable logistic regression analysis.

**Results:**

Among 511 patients, there were 125 patients in the pathological upgrading group (24.46%). The patients in the upgrading group were younger (47.68 ± 9.46 vs. 52.11 ± 7.02, *P* < 0.001), showed a lower proportion of menopausal women (38.40% vs. 53.02%, *P* = 0.0111), a lower proportion of HSIL (40.00% vs. 57.77%, *P* = 0.001), a higher rate of HPV-16/18 positive (25.60% vs. 17.36%, *P* = 0.011), a higher rate of contact bleeding (54.40% vs. 21.50%, *P* < 0.001), lower HDL levels (1.31 ± 0.29 vs. 1.37 ± 0.34 mmol/L, *P* = 0.002), higher neutrophil counts (median, 3.50 vs. 3.10 × 109/L, *P* = 0.001), higher red blood cell counts (4.01 ± 0.43 vs. 3.97 ± 0.47 × 1012/L, *P* = 0.002), higher platelet counts (204.84 ± 61.24 vs. 187.06 ± 73.66 × 109/L, *P* = 0.012), and a smaller platelet volume (median, 11.50 vs. 11.90 fL, *P* = 0.002).The multivariable logistic regression analysis showed that age (OR = 0.90, 95% CI: 0.86–0.94, *P* < 0.001), menopausal (OR = 2.68, 95% CI: 1.38–5.22, *P* = 0.004), contact bleeding (OR = 4.80, 95% CI: 2.91–7.91, *P* < 0.001), and mean platelet volume (OR = 0.83, 95% CI: 0.69–0.99, *P* = 0.038) were independently associated with pathological upgrading from CIN3 to CC after conical resection.

**Conclusion:**

Age, menopausal, contact bleeding, and mean platelet volume are risk factors of pathological upgrading from CIN3 to CC after conical resection, which could help identify high risk and susceptible patients of pathological upgrading to CC.

## Background

Cervical cancer (CC) is a malignancy originating in the transformation zone of the cervix, most commonly in squamous cells [1]. It is the second most common cancer in women worldwide and the third most common cause of female cancer mortality [1–3]. The types of CC include squamous cell carcinoma, invasive adenocarcinoma, small cell neuroendocrine carcinoma, and other rare histologic types [2, 4]. There is a causal link between persistent infection and oncogenic types of human papillomavirus (HPV), most commonly HPV-16 and HPV-18, which are sexually transmissible pathogens [1, 2, 5]. squamous intraepithelial lesion (SIL) category which encompasses a spectrum of squamous cell lesions starting from the precancerous lesions of low-grade SIL (LSIL) to high-grade SIL (HSIL), and ultimately invasive squamous cell carcinoma [1, 2, 5].. Of note, the regression of CIN can occur, with higher rates of regression seen with lower grades of CIN [6]. Cervical cytology screening in the United States of America has been associated with a > 70% reduction in CC incidence and mortality since the mid-1970s [7]. Women with abnormal cytology are usually referred for a biopsy [8, 9].

The timely detection of CIN3 is the cornerstone of CC screening guidelines [9, 10]. Indeed, CIN3 carries a risk of developing into in situ and invasive CC, but a CIN3 lesion at biopsy can also “hide” a more advanced lesion. Indeed, a biopsy takes only a small part of a lesion, and it is possible to miss the foci of most advanced pathologies. Hence, guidelines advocate the surgical removal of CIN3 lesions [2, 4, 9]. Indeed, a pathological upgrade to CC can be observed after surgery in about 10-11% of the patients [11, 12].

Still, determining in advance the patients in whom there is a greater probability of finding CC after CIN3 removal would be more conducive to improving patient outcomes by identifying patients requiring more urgent treatments. Wang et al. [12] reported that image quality of colposcopy images, atypical blood vessels, biopsy sampling method, and visible lesion area of the cervix were independently associated with CC after surgery for CIN3. Some of these factors are subjective and based on the physician’s experience and judgment. On the other hand, Jia et al. [11] reported more objective factors independently associated with CC after CIN3 removal, i.e., postmenopausal period ≥ 5 years, endocervical glandular involvement, endocervical curettage, and HPV16/18 infection.

Therefore, this study aims to investigate risk factors for postoperative pathological upgrading of cervical biopsy from CIN3 to CC after conical resection. The results could help early identify high-risk and susceptible individuals with CC and facilitates precise interventions.

## Methods

### Data collection

This retrospective study collected data from patients diagnosed with CIN3 by cervical biopsies at the author’s Hospital between January 2012 and December 2022. Inclusion criteria: (1) age 30–75 years old; (2) available HPV and cervical liquid-based cytological examination results; (3) definitive diagnosis of CIN3 by colposcopy examination results and cervical multi-point biopsy pathology, (4) underwent loop electrosurgical excision procedure (LEEP); (5) underwent hysterectomy. Exclusion criteria: (1) incomplete clinical data, (2) cervical biopsy pathology suspicious for or not excluding invasive CC, or (3) concurrent other malignancies. This study was approved by the medical ethics committee of the author’s Hospital. The requirement for informed consent was waived due to the retrospective nature of the study.

### Data collection and definitions

Clinical data were gathered through the electronic medical record system, including age, gravidity, parity, menopausal status, ThinPrep Cytology Test (TCT), high-risk HPV infection, involvement of glandular tissue, clinical symptoms, and preoperative laboratory test results. The TCT results were categorized into atypical squamous cells -cannot exclude high-grade squamous intraepithelial lesion (ASC-H), atypical glandular cells (AGC), high-grade squamous intraepithelial lesion (HSIL), negative for intraepithelial lesion or malignancy (NILM), atypical squamous cells (ASC), and low-grade squamous intraepithelial lesion (LSIL). ASC-H, AGC, and HSIL were grouped as the HSIL category, while NILM, ASC, and LSIL were grouped as the non-HSIL category. High-risk HPV infection was classified as HPV16/18 positive (considered positive if either HPV16 or HPV18 was detected, even in combination with other HPV types) or other high-risk types, excluding HPV16 and HPV18. The additional high-risk HPV types were 31, 33, 35, 39, 45, 51, 52, 56, 58, 59, 66, and 68. Cases with no relevant tests or HPV negativity were noted. Clinical symptoms encompassed contact bleeding (bleeding during sexual intercourse or vaginal bleeding), abnormal vaginal discharge (vaginal flow or abnormal discharge), or being asymptomatic with cervical lesions detected during routine physical examination. Laboratory indicators included high-density lipoprotein (HDL), albumin, lymphocyte count, monocyte count, neutrophil count, eosinophil count, red blood cell count, hemoglobin levels, hematocrit, red cell distribution width, platelet count, mean platelet volume, and fibrinogen.

### Outcomes

The primary outcome was the pathological results of patients after conical resection. The pathological findings were categorized into the pathological upgrading group if postoperative pathology indicated CC, while those with normal, inflammatory, or cervical precancerous lesions were classified into the pathological non-upgrading group.

### Statistical analysis

Statistical analysis was conducted using SPSS 22.0 (IBM Corp., Armonk, NY, USA). Missing values were handled through regression imputation. Continuous variables underwent normality testing; if they followed a normal distribution, an independent sample t-test was used for between-group comparisons. If the variables did not adhere to a normal distribution, the Wilcoxon-Mann-Whitney test was applied. Categorical variables were presented as n (%), and the chi-square test was used. Binary logistic regression analysis was used to explore independent factors associated with occult CC; variables with a P-value < 0.05 in the univariable analyses were included in the multivariable analysis. A two-sided P-value *P* < 0.05 was considered statistically significant.

## Results

A total of 534 patients met the inclusion criteria, but 23 were excluded due to incomplete clinical data, leaving 511 patients included. Among them, there were 125 patients in the pathological upgrading group (upgrading rate of 24.46%), and the non-upgrading group comprised 386 patients.

The characteristics of the patients are presented in Table 1. Compared with the non-upgrading group, the patients in the upgrading group were younger (47.68 ± 9.46 vs. 52.11 ± 7.02, *P* < 0.001), showed a lower proportion of menopausal women (38.40% vs. 53.02%, *P* = 0.0111), a lower proportion of HSIL (40.00% vs. 57.77%, *P* = 0.001), a higher rate of HPV-16/18 positive (25.60% vs. 17.36%, *P* = 0.011), a higher rate of contact bleeding (54.40% vs. 21.50%, *P* < 0.001), lower HDL levels (1.31 ± 0.29 vs. 1.37 ± 0.34 mmol/L, *P* = 0.002), higher neutrophil counts (median, 3.50 vs. 3.10 × 10^9^/L, *P* = 0.001), higher red blood cell counts (4.01 ± 0.43 vs. 3.97 ± 0.47 × 10^12^/L, *P* = 0.002), higher platelet counts (204.84 ± 61.24 vs. 187.06 ± 73.66 × 10^9^/L, *P* = 0.012), and a smaller platelet volume (median, 11.50 vs. 11.90 fL, *P* = 0.002).

**Table 1:** Characteristics of the patients

Clinical Information	Pathological upgrading group (*n* = 125)	Pathological non-upgrading group (*n* = 386)	P
Age (years), Mean ± SD	47.68 ± 9.46	52.11 ± 7.02	< 0.001
Gravidity, Median (IQR)	3.00 (2.00 4.00)	3.00 (2.00 4.00)	0.302
Parity, Median (IQR)	2.00 (1.00 2.00)	2.00 (1.00 2.00)	0.252
Menopausal status, n (%)			0.011
Yes	48 (38.40)	202 (53.02)	
No	73 (58.40)	179 (46.98)	
TCT, n (%)			0.001
HSIL	50 (40.00)	223 (57.77)	
Non-HSIL	33 (26.40)	83 (21.50)	
Unknown	42 (33.60)	80 (20.73)	
High-risk HPV infection, n (%)			0.011
HPV16/18 positive	32 (25.60)	67 (17.36)	
Other high-risk types	16 (12.80)	74 (19.17)	
Not checked	77 (61.60)	235 (60.88)	
HPV negative, n (%)	0	10 (2.59)	
Involvement of glandular tissue, n (%)			0.083
Yes	82 (69.81)	300 (77.92)	
No	43 (30.19)	85 (22.08)	
Clinical symptoms, n (%)			< 0.001
Contact bleeding	68 (54.40)	83 (21.50)	
Abnormal vaginal discharge	12 (9.60)	39 (10.10)	
Asymptomatic, detected during routine examination	45 (36.00)	264 (68.39)	
High-density lipoprotein (mmol/L)	1.31 ± 0.29	1.37 ± 0.34	0.002
Albumin (g/L), Median (IQR)	41.10 (39.60 44.30)	41.90 (39.41 44.40)	0.560
Lymphocyte count (10^9^/L), Median (IQR)	1.70 (1.35 2.20)	1.70 (1.40 2.00)	0.183
Monocyte count (10^9^/L), Median (IQR)	0.39 (0.30 0.51)	0.37 (0.30 0.47)	0.305
Neutrophil count (10^9^/L), Median (IQR)	3.50 (2.90 4.50)	3.10 (2.40 4.00)	0.001
Eosinophil count (10^9^/L), Median (IQR)	0.10 (0.06 0.17)	0.09 (0.06 0.15)	0.254
Red blood cell count (10^12^/L)	4.01 ± 0.43	3.97 ± 0.47	0.002
Hemoglobin (g/L), Median (IQR)	121.00 (109.00 127.00)	118.50 (112.00 126.00)	0.881
Hematocrit (%), Median (IQR)	36.10 (32.95 38.20)	36.00 (34.10 38.10)	0.567
Red cell distribution width, Median (IQR)	42.90 (40.70 46.55)	43.70 (41.58 46.23)	0.089
Platelet count (10^9^/L), Mean ± SD	204.84 ± 61.24	187.06 ± 73.66	0.012
Mean platelet volume (fl.), Median (IQR)	11.50 (10.60 12.30)	11.90 (11.00 12.90)	0.002
Fibrinogen (g/L), Median (IQR)	2.73 (2.32 3.11)	2.73 (2.39 3.06)	0.842

TCT: ThinPrep Cytology Test; HSIL: high-grade squamous intraepithelial lesion; HPV: human papillomavirus

The multivariable analysis of the factors associated with postoperative pathological upgrading to cervical cancer is shown in Table 2. Age (OR = 0.90, 95%CI: 0.86–0.94, *P* < 0.001), menopausal (OR = 2.68, 95%CI: 1.38–5.22, *P* = 0.004), contact bleeding (OR = 4.80, 95%CI: 2.91–7.91, *P* < 0.001), and mean platelet volume (OR = 0.83, 95%CI: 0.69–0.99, *P* = 0.038) were independently associated with a pathological upgrade to CC after surgery for CIN3 lesions.

**Table 2:** Multivariable logistic regression analysis for pathological upgrading of cervical biopsy from CIN3 to cancer after conical resection

Variables	Univariable model		Multivariable model	
	OR (95% CI)	P	OR (95% CI)	P
Age (years)	0.92 (0.90, 0.95)	< 0.001	0.90 (0.86, 0.94)	< 0.001
Gravidity	0.95 (0.84, 1.07)	0.400		
Parity	0.86 (0.68, 1.08)	0.201		
Menopausal Status				
Yes	0.58 (0.38, 0.88)	0.011	2.68 (1.38, 5.22)	0.004
No	ref		ref	
TCT				
HSIL	ref		ref	
Non-HSIL	1.77 (1.07, 2.94)	0.027	1.80 (0.99, 3.29)	0.055
Unknown	2.34 (1.44, 3.80)	0.001	1.61 (0.91, 2.89)	0.098
High-risk HPV infection				
HPV16/18 positive	ref		ref	
Other high-risk types	0.45 (0.23, 0.90)	0.023	0.54 (0.25, 1.15)	0.111
Not checked	0.69 (0.42, 1.12)	0.135	0.80 (0.44, 1.43)	0.443
HPV negative	-	-	-	-
Involvement of glandular tissue				
Yes	0.66 (0.41, 1.06)	0.084		
No	ref			
Clinical symptoms				
Contact bleeding	4.81 (3.06, 7.54)	< 0.001	4.80 (2.91, 7.91)	< 0.001
Abnormal vaginal discharge	1.81 (0.88, 3.71)	0.108	1.41 (0.63, 3.15)	0.406
Asymptomatic, detected during routine examination	ref		ref	
High-density lipoprotein	0.71 (0.37, 1.35)	0.298		
Albumin	0.98 (0.94, 1.03)	0.464		
Lymphocyte count	1.12 (0.87, 1.44)	0.389		
Monocyte count	1.19 (0.52, 2.74)	0.680		
Neutrophil count	1.16 (1.04, 1.29)	0.008	1.09 (0.96, 1.23)	0.189
Eosinophil count	1.77 (0.35, 8.90)	0.487		
Red blood cell count	1.26 (0.79, 2.02)	0.332		
Hemoglobin	1.00 (0.98, 1.01)	0.562		
Hematocrit	0.96 (0.91, 1.02)	0.210		
Red cell distribution width	1.02 (0.98, 1.05)	0.370		
Platelet count	1.00 (1.00, 1.01)	0.030	1.00 (1.00, 1.00)	0.714
Mean platelet volume	0.83 (0.71, 0.96)	0.014	0.83 (0.69, 0.99)	0.038
Fibrinogen	1.01 (0.83, 1.22)	0.928		

TCT: thinprep cytology test; HSIL: high-grade squamous intraepithelial lesion; HPV: human papillomavirus

## Discussion

This retrospective study investigated the factors associated with postoperative pathological upgrading to CC in patients with CIN3 following cervical biopsy. The results indicated that the pathological postoperative upgrading rate after CIN3 diagnosis at biopsy was high, at about 24.46%. Age, menopausal, contact bleeding, and mean platelet volume were independently associated with a higher probability of pathological upgrading to CC.

In the present study, the upgrading rate was 24.46%, higher than the 11% reported by Jia et al. [11] and the 6% reported by Fan et al. [13]. The discrepancy among studies cannot be explained by the data collected in the present study. Patient selection could play a role, as well as the criteria applied to select the patients for surgery (the present study did not include the patients who did not undergo surgery). It should be examined in future studies.

A study reported that image quality of colposcopy images, atypical blood vessels, biopsy sampling method, and visible lesion area of the cervix were independently associated with CC after surgery for CIN3 [12]. These factors above can be subjective and dependent upon the physician’s experience. Another study reported that a postmenopausal period ≥ 5 years, endocervical glandular involvement, endocervical curettage, and HPV16/18 infection were associated with upgrading to CC, which are more objective factors [14]. In the present study, age (OR = 0.90), menopausal (OR = 2.68), contact bleeding (OR = 4.80), and mean platelet volume (OR = 0.83) were independently associated with a pathological upgrade to CC after surgery for CIN3 lesions. The discrepancies among factors can be due to the available data in the retrospective studies.

Age is associated with the development of CC, and the peak age of incidence is 40–49 years, and most women develop CC before the age of 50 [5]. Accordingly, in the present study, the patients in the upgrading group were younger than those in the no-upgrading group. Still, such result must be considered with caution since cervical screening tend to decrease with age, which could affect the apparent incidence of CC [15]. A bimodal incidence of CC has been reported, with peaks at 30–39 and 60–69 years [16]. In the present study, very few women fell in the 60–69 age range. Additional studies are necessary to confirm the association of age with the upgrade of CIN3 at biopsy to CC at surgery.

Nevertheless, age and menopause are covariates. The decline in estrogen levels at menopause induces reductions in squamous epithelial cells, stromal blood vessels, and glycogen cell content. Hence, the sensitivity of acetic acid and iodine staining decreases [13, 17–19]. Suspicious lesions become difficult to evaluate and can be missed [20]. Indeed, Costa et al. [20] showed that after 50 years, the risk of missing a CC by colposcopy increases by 11 folds compared with patients < 30 years. Considering that menopause occurs in most Chinese women around 49–50 years of age [21–23] (80% are menopausal by 54 years [24]), menopause could be considered a risk factor for upgrading to CC after surgery for CIN3 lesions, as observed in the present study.

Vaginal bleeding or blood-stained vaginal discharge can be a sign of CC [25–28], but the risk of CC in women with postcoital bleeding is less clear [29]. In the present study, all women were diagnosed with CIN3 lesions. Vaginal bleeding is a sign that should prompt a medical consultation in the general population [27, 28]. In patients with a proven cervical lesion, additional signs such as vaginal bleeding could hint toward more severe lesions, as suggested by the present study.

The present study identified a decreased mean platelet volume as being associated with the pathological postoperative upgrading to CC in patients with CIN3 lesions at biopsy. Shen et al. [30] and Qin et al. [31] reported that a decreased mean platelet volume was associated with CC development and could even be used as a screening tool. In patients with CC, a decreased mean platelet volume is also associated with a poorer prognosis, indicating more aggressive or advanced disease [32, 33]. Platelets have complex relationships with cancer cells and contribute to tumor growth, angiogenesis, invasion, and dissemination [34, 35]. The mean platelet volume is associated with inflammatory conditions, including cancer [36, 37].It is suggested that the influence of HPV virus on blood cells is mainly reflected in the volume of platelets.

However, there were several limitations in this study. Firstly, this was a single-center study and with limited samples, which might have introduced selection bias. Secondly, this is a retrospective study, which is susceptible to investigator bias and recall bias. Thirdly, as an observational study, the available evidence was insufficient to definitively establish a causal relationship. Further studies were needed to investigate the potential risk factors of CIN3 upgrading to CC.

In conclusion, the pathological postoperative upgrading rate after CIN3 diagnosis at biopsy was high, at about 24.46%. Age, menopausal, contact bleeding, and mean platelet volume are objective parameters that could be used to identify patients with a higher probability of pathological upgrading to CC. Future studies should validate those factors to develop a nomogram that could be used to stratify the patients.

## Data Availability

All data generated or analysed during this study are included in this published article.

## Abbreviations

CC: Cervical cancer
CIN: Cervical intraepithelial neoplasia
LEEP: Loop electrosurgical excision procedure
TCT: Thinprep Cytology Test
HSIL: High-grade squamous intraepithelial lesion
NILM: Negative for intraepithelial lesion or malignancy
ASC: Atypical squamous cells
LSIL: Low-grade squamous intraepithelial lesion
HDL: High-density lipoprotein
